# Association between serum A/G ratio and stroke: data from NHANES 2009–2020

**DOI:** 10.3389/fnut.2025.1512165

**Published:** 2025-02-25

**Authors:** Xingle Tan, Cunming Lv, Chao Lu, Yanan Luo, Zhi-gang Mei

**Affiliations:** ^1^Third-Grade Pharmacological Laboratory on Chinese Medicine Approved by State Administration of Traditional Chinese Medicine, Medical College, China Three Gorges University, Yichang, China; ^2^The Second People’s Hospital of Yichang, China Three Gorges University, Yichang, China; ^3^The Key Laboratory of Hunan Province for Integrated Traditional Chinese and Western Medicine on Prevention and Treatment of Cardio-Cerebral Diseases, Hunan University of Chinese Medicine, Changsha, China

**Keywords:** NHANES, stroke, serum A/G, sex, index

## Abstract

**Background:**

The serum albumin-to-globulin ratio (A/G) has been widely used as a biomarker to assess inflammation, immunity, and nutritional status. However, relatively few studies have been conducted on the predictive value of serum A/G in stroke. Therefore, this study aimed to evaluate the correlation between serum A/G levels and stroke prognosis, to provide a new reference for risk assessment and management of stroke patients.

**Methods:**

Data were sourced from the National Health and Nutrition Examination Survey (NHANES) for 2009–2020. The study utilized questionnaire responses and 24-h dietary recall interviews. Participants were stratified by serum albumin/globulin (A/G) ratios into tertiles. Multivariable logistic regression, curve fitting, subgroup analyses, and interaction tests were conducted to assess the associations with serum A/G ratios.

**Results:**

Of the 82,298 participants initially considered, 52,119 had complete data and no history of stroke, albumin, or globulin deficiency, which were included in the analysis. We observed a decrease in stroke incidence with increasing A/G ratios. Higher A/G ratios were also associated with lower incidences of moderate exercise, diabetes, and coronary heart disease. The relationship between A/G ratios and stroke was moderated by covariates such as gender, hypertension, diabetes, smoking, and body mass index.

**Conclusion:**

In the US population, serum A/G ratios positively correlate with stroke incidence. Serum A/G could be a simple and economical marker for identifying stroke risk in the population, though further prospective studies are required to validate these findings.

## Introduction

Stroke, also known as cerebrovascular accident, is one of the leading causes of disability and death worldwide (1, 2). Stroke is further classified into two types, one being hemorrhagic stroke and the other ischaemic stroke. With the aging of the global population and improvement in the standard of living, the exposure to risk factors for stroke has increased significantly in developing countries. This has increased stroke-related morbidity and mortality in developing countries. In high-income countries, stroke epidemiology and outcomes continue to vary markedly by race, ethnicity, and geography (3). Over 90% of the stroke burden is attributable to modifiable risk factors, such as high blood pressure, obesity, etc.; 47 percent is attributable to behavioral risk factors such as smoking, sedentary lifestyle, etc. Controlling these behavioral and metabolic factors could prevent over three-quarters of strokes globally (4). Early identification of factors affecting a patient’s prognosis is essential for timely therapeutic intervention to improve outcomes (5–7). Therefore, it is critical in clinical practice to proactively identify pre-stroke conditions and their associated factors for primary intervention and prognostication (8).

Inflammatory, immune and nutritional factors are strongly associated with the etiology, development, and prognosis of stroke (9–11). A large body of evidence supports that various biomarkers such as Dietary Inflammatory Index (DII), Composite Dietary Antioxidant Index (CDAI) and Triglyceride-Glucose (TyG) are interrelated with increased risk of stroke (12–15). The serum albumin/globulin (A/G) ratio is a novel prognostic marker reflecting liver function, chronic kidney disease, systemic inflammation, and nutritional status (16, 17). Serum albumin is widely used as a surrogate for assessing nutritional status and is regarded as an independent prognostic factor for ischemic stroke (18–20). Meanwhile, serum globulin can assist in evaluating the severity of chronic inflammation (21, 22). Many studies have focused on the relationship between the A/G ratio and conditions such as cancer, cognitive decline, and various chronic diseases, suggesting its important role in the immune and inflammatory system (23, 24). A recent study found an association between serum A/G levels and acute ischemic stroke in a Chinese population (25). Unfortunately, this study did not take into account hemorrhagic stroke and its relationship with stroke incidence. Moreover, there are relatively few studies on the association between serum A/G and stroke in other countries. Therefore, further exploration of the relationship between serum A/G levels and stroke occurrence in different populations is important for improving prognosis and optimizing treatment strategies.

In this study, we aimed to assess the association between serum albumin/globulin (A/G) ratio and the prevalence of stroke, using data from the National Health and Nutrition Examination Survey (NHANES). This study utilized high-quality data from NHANES to provide useful information for clinical practice and thus provide a scientific basis for stroke prevention strategies.

## Materials and methods

### Participants

NHANES data has been an ongoing program since the early 1960s under the U.S. National Center for Health Statistics (NCHS). It employs a stratified multistage probability sampling technique to collect health and nutrition data (26). A key objective of NHANES is to extensively gather examination data from the U.S. elderly population to enhance our understanding of this demographic. The NCHS Ethics Review Board approved the NHANES survey protocol, and all participants provided written informed consent before their inclusion.

**Figure 1:**
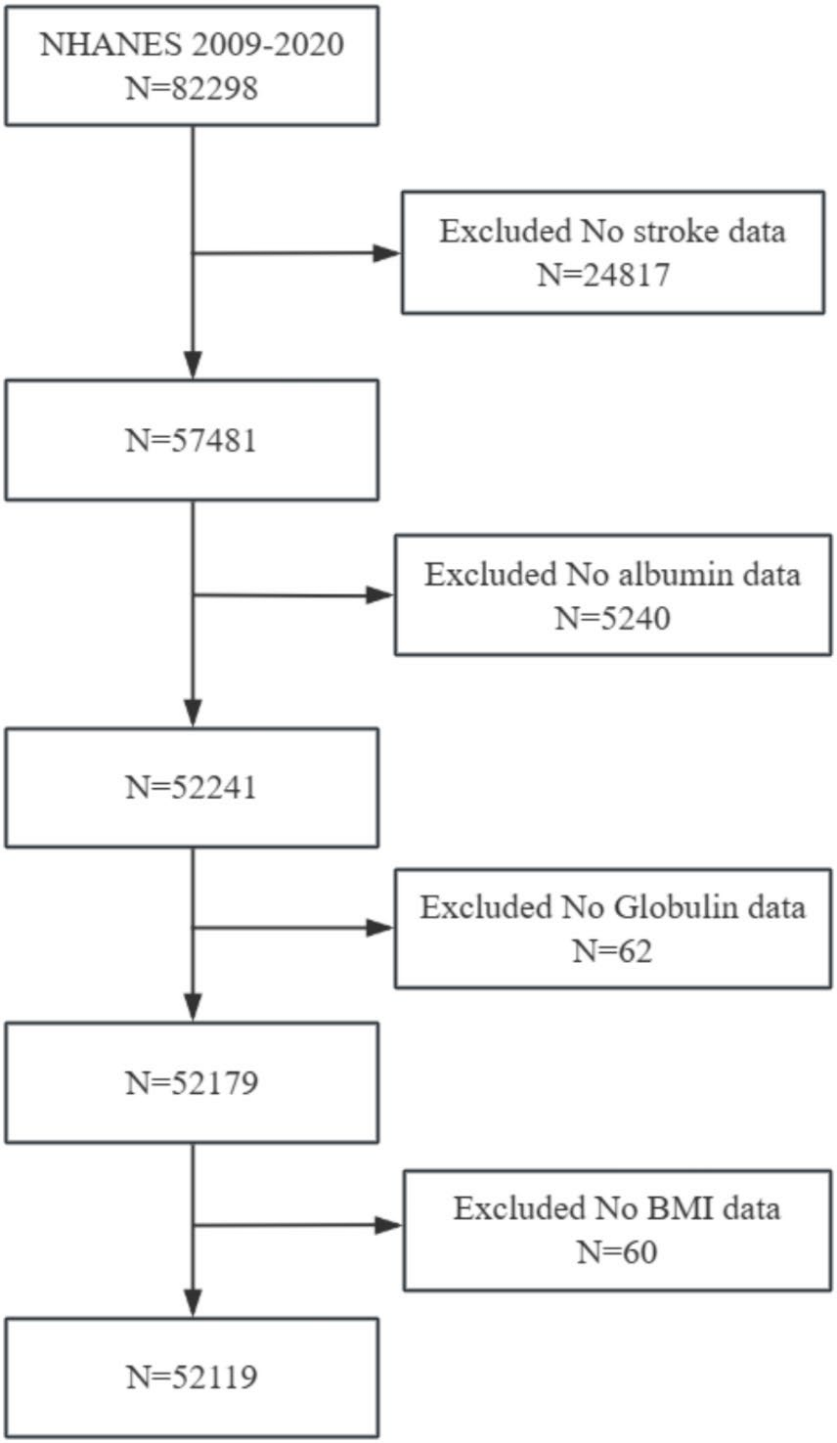
Flowchart of participants’ selection.

This study initially included cross-sectional data from 82,298 participants from the NHANES (2009–2020). The exclusion criteria were set as follows: (1) participants without stroke data (*n* = 82,298); (2) participants without albumin data (*n* = 5,240); (3) participants without globulin data (*n* = 62); (4) participants without BMI data (*n* = 60). A total of 52,119 participants were included in the analysis. A flowchart of participant enrollment is presented in Figure 1.

### Serum A/G

The method used by the DxC800 to measure albumin concentration is a two-color digital endpoint method. In the reaction, albumin binds to bromocresol purple (BCP) reagent to form a complex. The system monitors the change in absorbance at 600 nm. The change in absorbance is proportional to the concentration of albumin in the sample. Serum A/G = Albumin (g/L)/Globulin (g/L).

### Stroke

Participants were considered to have a history of stroke if they reported being previously informed by a doctor or other healthcare professional that they had a stroke (27, 28).

### Covariates

The baseline variables in this study included age, gender, ethnicity, diabetes, smoking status, body mass index (BMI), hypertension, income, education level, coronary heart disease, alcohol consumption habits, stroke history, and physical exercise. Ethnicity is divided into five categories Mexican American, Other Hispanic, Non-Hispanic White, Non-Hispanic Black and Other race. Being diagnosed with diabetes means that a doctor or other health professional has informed you that you have diabetes. Smoking is defined as subjects who smoked at least 100 cigarettes in their entire life. BMI stands for Body Mass Index, calculated as weight in kilograms divided by height in meters squared (kg/m^2). Normal weight is considered less than 25 kg/m^2, overweight is classified as having a BMI between 25 and 30 kg/m^2, and obesity is defined as having a BMI of 30 kg/m^2 or higher. Hypertension is defined as a condition characterized by a systolic blood pressure (SBP) over 140 mmHg and/or a diastolic blood pressure (DBP) over 90 mmHg. If a doctor or other health care professional has told you that you have high blood pressure and that you are recommended to take prescription medication, answering yes to both questions is a diagnosis of high blood pressure. Education level is divided into six categories: less than 9th grade, 9th-11th grade, high school graduation/GED or equivalent, partial college or AA degree, college graduation or higher, and others. Coronary heart disease is defined as having been told by a doctor or other health professional that you have congestive heart failure. A person is considered to be a drinker if he or she has had at least 12 alcoholic beverages of any type in his or her lifetime. Conditions such as hypertension, diabetes, coronary heart disease, and stroke were determined either through self-report or a doctor’s diagnosis. Details of all study variable measurement procedures are available at https://www.cdc.gov/nchs/nhanes/.

### Statistical analysis

Sample sizes were determined based on existing NHANES data. All statistical analyses were performed according to CDC guidelines using appropriate NHANES sampling weights and considered the complexity of the multistage cluster survey design. Continuous variables are summarized as means with standard errors (SE), and categorical variables are presented as percentages. Multivariate regression analysis was conducted based on tertiles of baseline characteristics or selected past studies. Model 1 was unadjusted. Model 2 adjusted for sex, age, and race. Model 3 adjusted for age, sex, ethnicity, diabetes, smoking habits, BMI, hypertension, income, education level, coronary heart disease, alcohol consumption, and physical exercise. Subgroup analyses were conducted according to sex, hypertension, diabetes, smoking status, race, and BMI categories, which were also considered potential effect modifiers, to examine whether there was a significant interaction between these covariates and A/G and stroke. In addition to this, we performed smoothed curve fitting and threshold effects to analyze and predict the non-linear relationship between A/G and stroke. All analyses were conducted using empowerstats 2.0.^1^ The significance level was set at *p* < 0.05.

## Results

### Baseline characteristics

This study involved 52,119 participants, comprising 25,216 males (48.38%) and 26,903 females (51.62%). Table 1 summarizes the baseline characteristics of participants stratified by tertiles of baseline serum A/G levels. Among all participants, 1,264 (2.43%) had a history of stroke, with a decreasing incidence correlating with higher A/G ratios. Participants in the highest A/G tertile were more likely to be male, Non-Hispanic White, non-smokers, have a normal BMI, be free of hypertension, have a higher income, and hold a college degree or higher. Higher A/G ratios were also associated with lower incidences of moderate exercise, diabetes, and coronary heart disease. Overall, the baseline data were stable.

**Table 1:** Baseline characteristics of the study population according to serum A/G levels tertiles.

Serum albumin-to-globulin ratio					
Variable	Total	Low	Middle	High	*P*
No.	52,119	17,163	17,138	17,818	
Age, mean ± SD		51.15 ± 17.06	49.75 ± 17.49	48.29 ± 18.10	*<0.001*
Gender, %					*<0.001*
Male	25,216 (48.38%)	6,234 (36.32%)	8,433 (49.21%)	10,549 (59.20%)	
Female	26,903 (51.62%)	10,929 (63.68%)	8,705 (50.79%)	7,269 (40.80%)	
Ethnicity, %					*<0.001*
Mexican American	7,181 (13.78%)	2,517 (14.67%)	2,608 (15.22%)	2056 (11.54%)	
Other Hispanic	5,553 (10.65%)	2022 (11.78%)	1861 (10.86%)	1,670 (9.37%)	
Non-Hispanic White	20,045 (38.46%)	4,018 (23.41%)	6,494 (37.89%)	9,533 (53.50%)	
Non-Hispanic Black	11,649 (22.35%)	6,170 (35.95%)	3,484 (20.33%)	1995 (11.20%)	
Other race	7,691 (14.76%)	2,436 (14.19%)	2,691 (15.70%)	2,564 (14.39%)	
Diabetes					*<0.001*
Yes	7,176 (13.77%)	3,212 (18.71%)	2,260 (13.19%)	1704 (9.56%)	
No	43,561 (83.58%)	13,427 (78.23%)	14,379 (83.90%)	15,755 (88.42%)	
Borderline	1,349 (2.59%)	506 (2.95%)	486 (2.84%)	357 (2.00%)	
Other	33 (0.01%)	18 (0.10%)	13 (0.08%)	2 (0.01%)	
Smoke					*<0.001*
Yes	22,358 (42.90%)	6,764 (39.41%)	7,341 (42.83%)	8,253 (46.32%)	
No	29,723 (57.03%)	10,373 (60.44%)	9,789 (57.12%)	9,561 (53.66%)	
Other	38 (0.07%)	26 (0.15%)	8 (0.05%)	4 (0.02%)	
BMI					*<0.001*
Normal weight < 25	17,029 (32.67%)	4,017 (23.87%)	5,501 (32.44%)	7,511 (42.53%)	
Over weight (25, 30)	17,270 (33.14%)	5,009 (29.76%)	5,890 (34.73%)	6,371 (36.07%)	
Obese ≥30	17,152 (32.89%)	7,805 (46.37%)	5,568 (32.83%)	3,779 (21.40%)	
High blood pressure					*<0.001*
Yes	19,236 (36.91%)	7,533 (43.89%)	6,117 (35.69%)	5,586 (31.35%)	
No	32,819 (62.97%)	9,612 (56.00%)	10,990 (64.13%)	12,217 (68.57%)	
Other	64 (0.02%)	18 (0.10%)	31 (0.18%)	15 (0.08%)	
Income					*<0.001*
<= 1.30	16,886 (32.40%)	6,397 (39.66%)	5,556 (34.20%)	4,933 (28.91%)	
1.30–1.85	7,079 (13.58%)	2,394 (14.84%)	2,403 (14.79%)	2,282 (13.37%)	
> 1.85	24,076 (46.19%)	6,774 (42.00%)	7,825 (48.17%)	9,477 (55.54%)	
Other	1,197 (2.30%)	565 (3.50%)	262 (2.85%)	370 (2.17%)	
Education					*<0.001*
Less than 9th grade	4,907 (9.41%)	2010 (11.71%)	1,659 (9.68%)	1,238 (6.95%)	
9th–11th grade	6,619 (12.70%)	2,545 (14.83%)	2,182 (12.73%)	1892 (10.62%)	
High school graduate/GED or equivalent	11,705 (22.46%)	3,974 (23.15%)	3,942 (23.00%)	3,816 (21.42%)	
Some college or AA degree	16,054 (30.80%)	5,367 (31.27%)	5,169 (30.16%)	5,518 (30.97%)	
College graduate or above	12,753 (24.47%)	3,237 (18.86%)	4,170 (24.33%)	5,346 (30.00%)	
Other	54 (0.05%)	30 (0.18%)	16 (0.09%)	8 (0.04%)	
Coronary Heart Disease					*0.012*
Yes	2,139 (4.10%)	755 (4.40%)	689 (4.02%)	695 (3.90%)	
No	49,799 (95.55%)	16,336 (95.18%)	16,386 (95.61%)	17,077 (95.84%)	
Other	181 (0.34%)	72 (0.42%)	63 (0.37%)	46 (0.26%)	
Alcohol use, %		3.87 ± 34.24	3.97 ± 34.83	3.46 ± 27.12	*<0.001*
Exercise		74.25 ± 367.15	64.39 ± 59.68	69.71 ± 165.38	*<0.001*
Stroke					*<0.001*
Yes	1,264 (2.43%)	955 (5.56%)	628 (3.66%)	541 (3.04%)	
No	49,947 (95.83%)	16,187 (94.31%)	16,493 (96.24%)	17,267 (96.91%)	
Other	48 (1.76%)	21 (0.12%)	17 (0.10%)	10 (0.06%)	

All italicised *P*’s are *p*-values, meaning trends.

### Association between stroke and serum A/G

Table 2 lists results from multivariable logistic regression analyses exploring the relationship between stroke and variables such as A/G, diabetes, hypertension, BMI, income, physical exercise, and education level. In Models 1 and 2, significant positive correlations were observed between stroke, A/G, and diabetes (*p < 0.05*). In Model 3, this positive correlation remained statistically significant within the highest tertile of A/G (OR = 0.01; 95% CI, 0.00–0.02; *p = 0.0302*), with a 1% increase in stroke incidence corresponding to an increase in the highest A/G tertile. Across all three models, stroke was positively correlated with higher tertiles of hypertension, income, and education, showing significant differences.

**Table 2:** Multivariate logistic regression model of stroke with A/G, diabetes, hypertension, and education level.

	β/OR^a^ (95% CI^b^), *P* value		
	Model 1^c^	Model 2^d^	Model 3^e^
A/G			
Tertile 1	Reference	Reference	Reference
Tertile 2	0.02 (0.01,0.02) *<0.0001*	0.01 (0.01,0.02) *<0.0001*	0.01(−0.00,0.02) *0.1581*
Tertile 3	0.02 (0.01,0.03) *<0.0001*	0.01 (0.01,0.02) *<0.0001*	0.01 (0.00,0.02) *0.0302*
Diabetes			
Tertile 1	Reference	Reference	Reference
Tertile 2	0.06 (0.05,0.07) *<0.0001*	0.04 (0.03,0.04) *<0.0001*	0.01(−0.00,0.02) *0.1475*
Tertile 3	0.03 (0.02,0.05) *<0.0001*	0.03 (0.01,0.05) *0.0006*	−0.02(−0.05,0.00) *0.0700*
Tertile 4	0.39 (0.29,0.49) *<0.0001*	0.37 (0.27,0.47) *<0.0001*	0.03(−0.18,0.24) *0.7925*
Hypertension			
Tertile 1	Reference	Reference	Reference
Tertile 2	0.06 (0.06,0.07) *<0.0001*	0.04 (0.03,0.05) *<0.0001*	0.02 (0.01,0.03) *<0.0001*
Tertile 3	0.01(−0.06,0.08) *0.7482*	0.00(−0.07,0.07) *0.9936*	0.04(−0.13,0.21) *0.6762*
Education			
Tertile 1	Reference	Reference	Reference
Tertile 2	−0.01(−0.02,0.00) *0.2937*	−0.01(−0.02,0.00) *0.1279*	−0.04(−0.07,-0.02) *0.0013*
Tertile 3	−0.00(−0.01,0.01) *0.6378*	−0.00(−0.01,0.01) *0.5255*	−0.06(−0.08,-0.04) *<0.0001*
Tertile 4	0.01 (0.00,0.02) *0.0132*	0.01(−0.00,0.02) *0.1928*	−0.05(−0.07,-0.02) *<0.0001*
Tertile 5	0.02 (0.01,0.03) *<0.0001*	0.02 (0.01,0.03) *<0.0001*	−0.04(−0.06,-0.02) *0.0005*

For A/G ratios, Tertile 1 represents the lowest third of the population with A/G ratios and Tertile 3 represents the highest third of the population with A/G ratios. For diabetes, Tertile 1 represents the third of the population with the lowest risk of diabetes, while Tertile 3 represents the third of the population with the highest risk of diabetes. The same is true for hypertension. For education level, Tertile 1–5 represents less than 9th grade, 9th-11th grade, high school graduate/GED or equivalent, some college or AA degree, college graduate or above.

^aβ/OR: effect size/odd ratio.^

^b95% CI: 95% confidence interval.^

^cModel 1: no covariates were adjusted.^

^dModel 2: adjusted for gender, age, and race.^

^eModel 3: adjusted for gender, age, race, A/G, diabetes, hypertension, education level.^

All italicised *P*’s are *p*-values, meaning trends.

### Subgroup analysis

To assess the consistency of the relationship between A/G ratios and stroke across the general population and to identify potential differences across demographic subgroups, we conducted subgroup analyses and interaction tests stratified by gender, hypertension, diabetes, smoking, ethnicity, and BMI. The results indicated an inconsistent relationship between the two. As shown in Table 3, significant interactions were found between A/G score and gender, and smoking (*P* for interaction *<0.05*), but no interaction effects were observed for hypertension, diabetes, ethnicity, or BMI. The relationship between A/G and stroke showed significant positive correlations across the stratified subgroups of gender, hypertension, diabetes, smoking, and BMI (*p < 0.05*). However, in the ethnicity-stratified subgroup, a statistically significant association was observed only among non-Mexican, non-American populations (*β* = 0.65; 95% CI, 0.43–1.00; *p = 0.0477*). Our findings suggest that the association between A/G ratios and stroke depends on gender, hypertension, diabetes, smoking, and BMI conditions.

**Table 3:** Subgroup analysis for the association between A/G ratios and stroke.

A/G	A/G score		Severe A/G	
	β (95% CI), *p* for trend	*P* for interaction	OR (95% CI), *p* for trend	*P* for interaction
Gender				
Male	0.40 (0.34,0.46) *<0.0001*	*<0.0001*	0.68 (0.43,1.08) *0.1031*	*0.2898*
Female	0.47 (0.40,0.55) *<0.0001*		2.17 (0.11,41.95) *0.6069*	
Subgroup analysis stratified by hypertension				
Yes	0.69 (0.61,0.79) *<0.0001*	*0.1629*	1.26 (0.82,1.96) *0.2942*	*0.0048*
No	0.11 (0.09,0.13) *<0.0001*		0.03 (0.00,0.55) *0.0172*	
Subgroup analysis stratified by diabetes				
Yes	0.66 (0.55,0.81) *<0.0001*	*0.0829*	1.36 (0.68,2.70) *0.3834*	*0.4273*
No	0.22 (0.19,0.25) *<0.0001*		0.14 (0.01,1.76) *0.1292*	
Smoke				
Yes	0.44 (0.39,0.51) *<0.0001*	*0.0215*	0.70 (0.45,1.09) *0.1145*	*0.1078*
No	0.25 (0.22,0.30) *<0.0001*		0.15 (0.02,1.21) *0.0756*	
Race				
Mexican American	0.72 (0.49,1.05) *0.0849*	*0.2167*	1.35 (0.40,4.52) *0.6249*	*0.5084*
Other Hispanic	0.65 (0.43,1.00) *0.0477*		1.33 (0.36,4.88) *0.6683*	
Non-Hispanic White	1.28 (0.99,1.65) *0.0589*		1.75 (0.61,5.02) *0.3014*	
Non-Hispanic Black	1.04 (0.73,1.47) *0.8325*		1.53 (0.44,5.28) *0.5004*	
Other race	0.73 (0.51,1.05) *0.0870*		1.07 (0.29,3.97) *0.9148*	
BMI				
Normal weight < 25	0.46 (0.38,0.57) *<0.0001*	*0.1400*	0.99 (0.52,1.92) *0.9868*	*0.0447*
Overweight (25, 30)	0.54 (0.45,0.66) *<0.0001*		1.25 (0.08,19.64) *0.8733*	
Obese ≥30	0.62 (0.50,0.77) *<0.0001*		5.76 (0.31,105.62) *0.2382*	

All italicised *P*’s are *p*-values, meaning trends.

Specifically, interaction effects were noted for hypertension (*p = 0.0048*) and BMI (*p = 0.0447*) in this relationship. Within the hypertension-stratified subgroup, each unit increase in A/G was associated with a 3% increase in stroke incidence among participants without hypertension (OR = 0.03; 95% CI, 0.00–0.55; *p = 0.0172*). No significant differences were observed in the interaction tests for gender, diabetes, smoking, and ethnicity (*P* for interaction *>0.05*).

**Figure 2:**
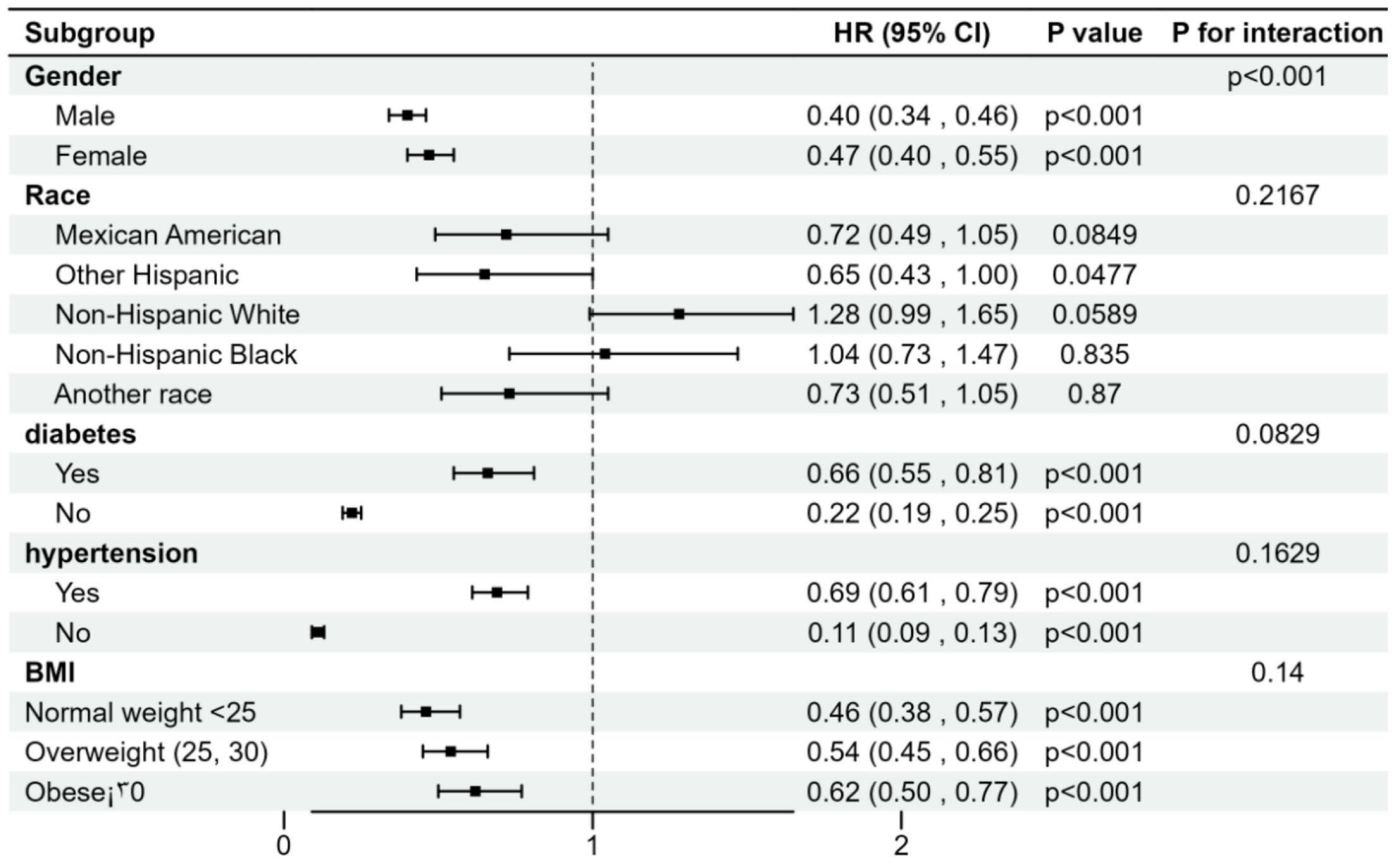
Forest plots of stratified analyses of A/G ratio and stroke.

The effect of A/G ratio on the incidence of stroke was stratified by sex, race, history of diabetes, history of hypertension, and body mass index, and risk ratio analyses and interaction tests were performed. Our results indicate that these associations were not consistent, as shown in Figure 2, where we detected a significant interaction for sex (*p* < 0.05) and no significant interaction for the rest. The association between A/G ratio and stroke was negatively associated with risk ratios for sex, other Hispanic origin, history of diabetes and hypertension, and BMI subgroups. In the association between A/G ratio and stroke, patients with diabetes and hypertension had a higher risk than non-diabetics and non-hypertensives, while the risk ratio also increased with increasing BMI.

### Curve fitting threshold analysis

**Figure 3:**
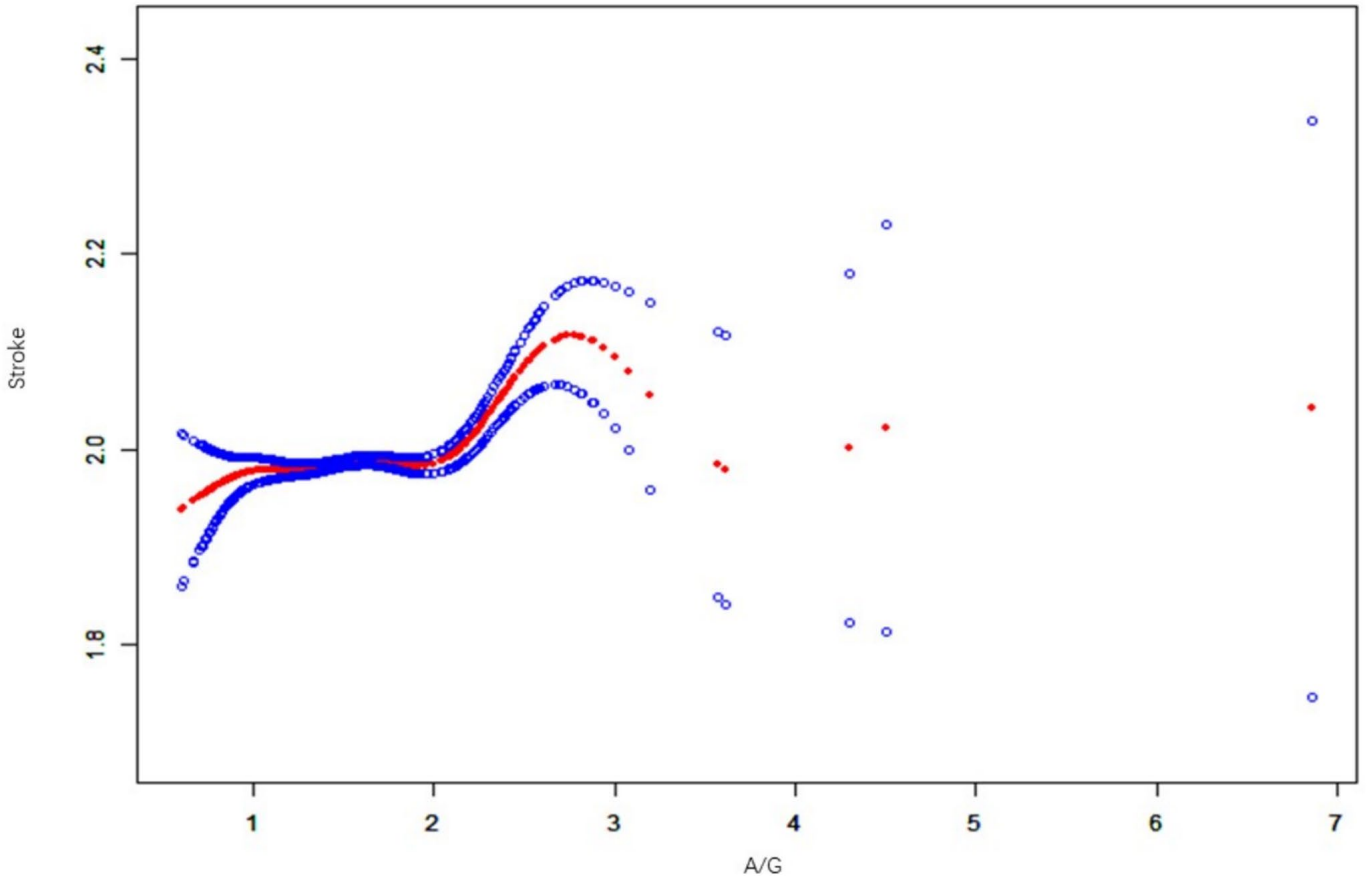
A non-linear relationship between A/G and stroke by the generalized additive model. Data includes: β (95% CI) *p*-value/OR (95% CI) *p*-value; outcome variable: stroke; exposure variable: A/G ratio; adjusted variables: alcohol, smoking, income, hypertension, exercise, BMI, diabetes, sex, age, race, education, coronary artery disease.

Through curve-fitting threshold analysis, we identified a nonlinear relationship between A/G ratios and stroke incidence. The threshold (K) for the A/G ratio was determined to be 1.88 g/L. To the left of this threshold, no statistically significant relationship was found between A/G and stroke (*β* = 0.01; 95% CI, −0.00-0.03; *p* = 0.1309). However, to the right of the threshold, a positive correlation was observed between A/G and stroke (β = 0.06; 95% CI, 0.03–0.09; *p* = 0.0002). The log-likelihood ratio test, before and after adjustment for covariates, yielded a *p*-value of 0.017 (see Figure 3).

**Figure 4:**
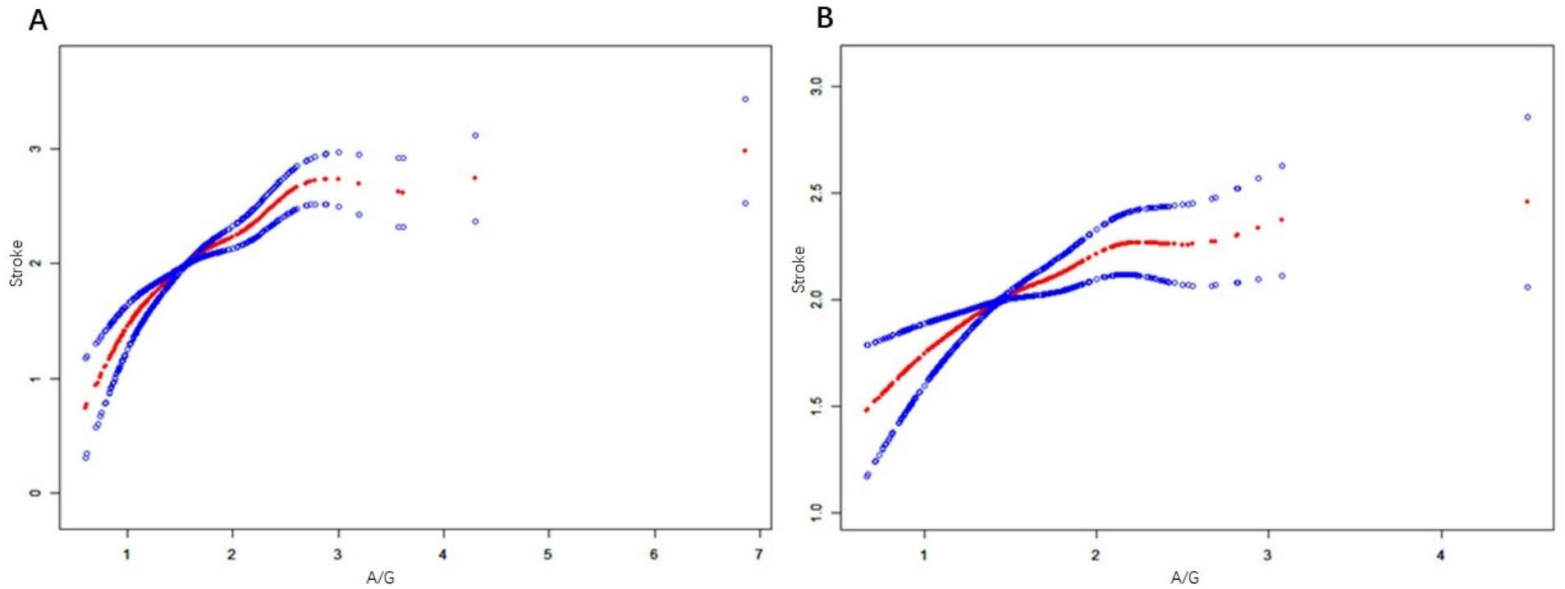
**(A)** is a generalized additive model that establishes the nonlinear relationship between A/G and stroke in men. **(B)** is a generalized additive model that establishes the nonlinear relationship between A/G and Stroke in women. Data includes: β (95% CI) *p*-value/OR (95% CI) *p*-value; Outcome variable: STROKE; Exposure variable: A/G ratio; adjusted variables: alcohol, smoking, income, hypertension, exercise, BMI, diabetes, sex, age, race, education, coronary artery disease.

We also explored whether there was a potential sex differences between the A/G ratio and stroke prevalence. In men, the association between A/G and stroke was nonlinear and positive. The threshold (K) for the A/G ratio was determined to be 1.55 g/L. To the left of this threshold, there was a statistically significant relationship between A/G and stroke (*β* = 0.21; 95% CI, 0.06–0.35; *p* = 0.0048). However, to the right of the threshold, a positive association between A/G and stroke was observed (*β* = 0.12; 95% CI, 0.05–0.18; *p* = 0.0003). The log-likelihood ratio test yielded a *p* value of 0.059 before and after adjusting for covariates. In women, the association between A/G and stroke was also nonlinear and positive. The threshold (K) for the A/G ratio was determined to be 1.48 g/L. To the left of this threshold, there was a statistically significant relationship between A/G and stroke (*β* = 0.26; 95% CI, 0.06–0.46; *p* = 0.0098). However, to the right of the threshold, a positive association was observed between A/G and stroke (*β* = 0.13; 95% CI, 0.03–0.23; *p* = 0.0112). The log-likelihood ratio test yielded a *p* value of 0.024 before and after adjusting for covariates (see Figure 4).

## Discussion

In recent years, the role of the albumin-to-globulin ratio (A/G ratio) in stroke research has gained increasing attention. Current studies suggest that the A/G ratio, as a simple and cost-effective marker, can reflect a patient’s nutritional status, inflammation level, and immune function. Wang et al. (25), in a study of acute ischemic stroke (AIS) patients in a Chinese population, found that a lower A/G ratio was significantly associated with poorer functional outcomes and higher all-cause mortality. These findings support the A/G ratio as a potential predictor of stroke risk. However, most of these studies have been limited to specific populations and regions, lacking broader validation.

This study aimed to explore the relationship between the A/G ratio and stroke risk in the general U.S. population by analyzing NHANES data from 2009–2020. The results revealed a nonlinear relationship between the A/G ratio and stroke, with a significant positive correlation even after adjusting for multiple confounding factors. Compared to other serum markers, the association between the A/G ratio and stroke was more pronounced (29).

Specifically, our study found that individuals with a lower A/G ratio had a higher incidence of stroke, consistent with the findings of Wang et al. (25) in AIS patients in China. However, unlike previous studies, we also found that a higher A/G ratio may be associated with increased stroke risk in certain cases. This discrepancy may be due to sample characteristics, duration of follow-up, and other potential confounding factors.

Notably, the A/G ratio is not only relevant for stroke prognosis but has also attracted attention for its role in other diseases. For instance, Ibrahim et al. (30) found that cardiovascular event patients with lower serum A/G ratios at admission showed poorer functional outcomes after three months of follow-up. This finding further supports the broad applicability of the A/G ratio as a prognostic marker. However, for specific diseases such as cognitive decline (31), the exact mechanism of the A/G ratio remains unclear, suggesting that its role may differ across various disease contexts.

Our findings offer new insights into the A/G ratio as a biomarker for stroke risk. By using a large, nationally representative sample and adjusting for various confounders, we further validated the association between the A/G ratio and stroke risk, highlighting its potential as a comprehensive marker for nutritional status, inflammation, and immune function. This integrated approach can help clinicians more accurately identify high-risk stroke populations and develop more targeted prevention strategies.

Despite the strengths of this study, such as the large sample size and multivariable adjustments, there are certain limitations. First, due to the cross-sectional design, causality cannot be established. Second, the sample is limited to the U.S. population, and the generalizability of the findings needs further validation in other regions and populations with different environmental and genetic backgrounds. Future studies should consider including more potential confounding factors, such as socioeconomic status and environmental exposure, for a more comprehensive understanding of the relationship between the A/G ratio and stroke.

The serum A/G ratio, as a simple and cost-effective marker for assessing stroke risk, has significant clinical value (32, 33). The serum albumin-to-globulin ratio (A/G ratio) is strongly associated with the occurrence and prognosis of stroke. Lower A/G ratios are associated with poor functional outcomes and all-cause mortality in patients with acute ischaemic stroke. However, although the correlation between the A/G ratio and stroke has been identified, there is insufficient evidence to suggest that adjustment of treatment strategies based on the AG ratio significantly improves patient prognosis. Therefore, clinical treatment should take into account the patient’s overall condition, including blood pressure, blood glucose, lipids, lifestyle, etc. The A/G ratio can be used as a reference indicator for evaluating the patient’s health status but should not be used alone as a basis for treatment decisions. In conclusion, the A/G ratio has a certain reference value in the assessment of stroke patients, but multiple clinical factors should be taken into account when formulating treatment plans to avoid making treatment decisions based on a single indicator alone.

## Conclusion

Extensive validation of A/G as a stroke biomarker: Our study extends previous findings, particularly those based on specific populations (e.g., Chinese), by providing evidence from a large nationally representative sample (NHANES 2009–2020). This reinforces the generalisability of A/G as a stroke biomarker in diverse populations, particularly in the US context. Identification of a non-linear relationship: This study highlights a non-linear relationship between A/G ratios and stroke incidence, suggesting that above a certain threshold, higher A/G ratios may be associated with an increased risk of stroke. This nuanced finding adds depth to existing studies that have only observed direct correlations, providing a more complex view of how the A/G ratio affects stroke risk. Gender and Other Subgroup Differences: This study revealed how the relationship between the A/G ratio and stroke risk varied across demographic subgroups, including gender, smoking status, and hypertension. This subgroup analysis suggests that clinicians should consider these factors when using A/G ratios as potential markers of stroke risk, thereby guiding a more personalized assessment of stroke risk. Comprehensive Multivariable Adjustment: Our study considered several potentially confounding variables, such as age, diabetes, hypertension, BMI, and others. Doing so provides a clearer and more robust understanding of the potential of the A/G ratio as a reliable marker of stroke risk in clinical practice. Pathophysiological insights and public health implications: By linking the A/G ratio to stroke risk, our study provides important clues to understanding how systemic inflammation, immune function, and nutritional status contribute to stroke occurrence. This positions A/G as a simple, cost-effective biomarker that can be easily integrated into routine clinical practice to identify at-risk individuals and inform prevention strategies.

Overall, our study significantly advances the literature by providing a comprehensive analysis of A/G as a marker of stroke risk, highlighting its potential use in the clinical setting, while also identifying the need for further prospective studies to validate these findings.

## Data Availability

The original contributions presented in the study are included in the article/supplementary material, further inquiries can be directed to the corresponding authors.

## Footnotes

^1^ https://www.empowerstats.net/cn/index.php
